# BK channel β1 and β4 auxiliary subunits exert opposite influences on escalated ethanol drinking in dependent mice

**DOI:** 10.3389/fnint.2013.00105

**Published:** 2013-12-30

**Authors:** Max Kreifeldt, David Le, Steven N. Treistman, George F. Koob, Candice Contet

**Affiliations:** ^1^Committee on the Neurobiology of Addictive Disorders, The Scripps Research InstituteLa Jolla, San Diego, CA, USA; ^2^Institute of Neurobiology, University of Puerto RicoSan Juan, PR, USA

**Keywords:** alcohol, knockout mice, two-bottle choice, vapor chambers, dependence

## Abstract

Large conductance calcium-activated potassium (BK) channels play a key role in the control of neuronal activity. Ethanol is a potent activator of BK channel gating, but how this action may impact ethanol drinking still remains poorly understood. Auxiliary β subunits are known to modulate ethanol-induced potentiation of BK currents. In the present study, we investigated whether BK β1 and β4 subunits influence voluntary ethanol consumption using knockout (KO) mice. In a first experiment, mice were first subjected to continuous two-bottle choice (2BC) and were then switched to intermittent 2BC, which progressively increased ethanol intake as previously described in wildtype mice. BK β1 or β4 subunit deficiency did not affect ethanol self-administration under either schedule of access. In a second experiment, mice were first trained to drink ethanol in a limited-access 2BC paradigm. BK β1 or β4 deletion did not affect baseline consumption. Weeks of 2BC were then alternated with weeks of chronic intermittent ethanol (CIE) or air inhalation. As expected, a gradual escalation of ethanol drinking was observed in dependent wildtype mice, while intake remained stable in non-dependent wildtype mice. However, CIE exposure only produced a mild augmentation of ethanol consumption in BK β4 KO mice. Conversely, ethanol drinking increased after fewer CIE cycles in BK β1 KO mice than in wildtype mice. In conclusion, BK β1 or β4 did not influence voluntary ethanol drinking in non-dependent mice, regardless of the pattern of access to ethanol. However, deletion of BK β4 attenuated, while deletion of BK β1 accelerated, the escalation of ethanol drinking during withdrawal from CIE. Our data suggest that BK β1 and β4 subunits have an opposite influence on the negative reinforcing properties of ethanol withdrawal. Modulating the expression, distribution or interactions of BK channel auxiliary subunits may therefore represent a novel avenue for the treatment of alcoholism.

## Introduction

One of the well-established molecular targets of ethanol is the large conductance calcium-activated (BK) potassium channel. BK channels are widely distributed throughout the body but particularly abundant in the brain, with high levels of expression in the cortex, limbic system, basal ganglia, thalamus and cerebellum (Chang et al., 1997; Sausbier et al., 2006). They play a key role in several aspects of neuronal physiology, including neurotransmitter release, action potential repolarization, firing patterns, and dendritic excitability [see Faber and Sah (2003) for review]. Each native BK channel exists as an assembly of four pore-forming α subunits, potentially associated with four auxiliary β subunits. The α subunit is characterized by an exceptionally high conductance for potassium ions (200–300 pS) and is activated both by membrane depolarization and intracellular Ca^2+^ elevation (Marty, 1981; Butler et al., 1993).

Ethanol potently affects BK channel gating by increasing the time spent in the open state, without altering conductance or ion selectivity (Dopico et al., 1996). The behavioral significance of BK channel potentiation by ethanol is however unclear. In *C. elegans*, loss-of-function mutations in the gene encoding BK α subunit led to resistance to ethanol intoxication, while gain-of-function mutations mimicked intoxication (Davies et al., 2003). In contrast, in *D. melanogaster*, similar manipulations pointed to a role of BK α subunit in rapid tolerance to the sedative effect of ethanol (Cowmeadow et al., 2005, 2006).

The association of auxiliary β subunits modifies the biophysical properties, pharmacology and trafficking of BK α [see Torres et al. (2007); Sun et al. (2012) for reviews]. In particular, β subunits modulate the sensitivity of BK currents to ethanol-induced potentiation (Martin et al., 2004; Feinberg-Zadek and Treistman, 2007). Interestingly, deletion of BK β4, the most prominent β subunit in the brain (Behrens et al., 2000; Brenner et al., 2000), in mice triggered rapid tolerance to the effects of ethanol on BK currents, action potential patterning and locomotion, while increasing limited-access ethanol drinking (Martin et al., 2008). The influence of BK β1, which shows low levels of expression in the brain but confers resistance to ethanol-induced activation of BK currents (Martin et al., 2004; Feinberg-Zadek and Treistman, 2007), on the behavioral effects of ethanol remains to be explored.

In the present study we sought to determine whether BK β1 and β4 subunits modulate voluntary ethanol consumption. To that end, we tested knockout (KO) mice for the BK β1 and β4 subunits in several paradigms of two-bottle choice (2BC) drinking.

## Materials and methods

### Animals

BK β1 and β4 KO mice were generated by homologous recombination (Brenner et al., 2000, 2005) and fully backcrossed on C57BL/6J background. BK β1 and β4 wildtype (WT), heterozygous (Het) and KO littermates were bred at The Scripps Research Institute in a temperature (22°C) and humidity (50%) controlled vivarium. Only group-housed males were used in experiments. Mice were at least 10 weeks old when testing started. They were first acclimated for 1 week to reverse light cycle (12-h light/dark cycle, lights on at 10:15 PM). Mice were transferred to individual caging in a dedicated room three days before testing started and remained single-housed for the whole duration of the experiment. Water (acidified) and food (standard rodent chow, Harlan Teklad, Frederick, MD, USA) were available *ad libitum* at all times. All procedures were carried out in accordance with the National Institutes of Health *Guide for the Care and Use of Laboratory Animals* and were approved by The Scripps Research Institute Institutional Animal Care and Use Committee.

### Two-bottle choice ethanol drinking paradigms

All drinking experiments were conducted in the mouse home cage. Fifty mL conical tubes fitted with a size 6 rubber stopper and 2.5″ stainless steel straight ball tube (Ancare, Bellmore, NY) were used as drinking bottles. Ethanol solutions were prepared with 95% ethanol (Pharmco-AAPER, Brookfield, CT) and acidified water. Mice were weighed once a week in order to calculate ethanol intake in g/kg.

#### Experiment 1: continuous and intermittent access

BK β1 and β4 WT, Het and KO mice were first offered continuous access to two bottles, one containing ethanol (20% w:v) and one containing water, for two consecutive weeks. Bottles were weighed and their positions inverted every day, Monday through Saturday, at 1:00 PM. Mice were then offered intermittent 24-h access to ethanol for two consecutive weeks. On Tuesdays, Thursdays and Saturdays, the bottle of ethanol was replaced with a bottle of water. On Mondays, Wednesdays and Fridays, the bottle of ethanol was reintroduced, in the position opposite to its last presentation. Bottle weights were recorded every day, Monday through Saturday, at 1:00 PM. This paradigm was adapted from previous publications (Hwa et al., 2011; Melendez, 2011). Under these experimental conditions, C57BL/6J male mice (Jackson Laboratories, Sacramento, CA) offered intermittent access had intoxicating blood alcohol levels (BALs) when tail blood was sampled 1 h after reintroduction of the ethanol bottle (Supplementary Figure S1). In contrast, BALs were below the intoxication threshold (80 mg/dl) in mice having continuous access to ethanol, as well as in mice having intermittent access when tail blood was sampled 24 h after reintroduction of the ethanol bottle (Supplementary Figure S1).

#### Experiment 2: limited access and dependence induction

Because of the limited number of available ethanol vapor chambers, this experiment was completed by staggering several cohorts of mice (6 cohorts for BK β1 and 5 cohorts for BK β4) over a total duration of 18 months. Importantly, each cohort contained an equivalent number of age-matched WT and KO mice and treatment conditions were counterbalanced for both genotypes within each cohort.

BK β1 and β4 WT and KO mice were offered access to two bottles, one containing ethanol (15% v:v) and one containing water, for 2 h per day from 10:00 AM to 12:00 PM, 5 days a week (Monday through Friday). Bottles were weighed and their positions inverted daily. A standard home cage water bottle was available the rest of the time. Once ethanol intake stabilized (typically after 2 weeks), mice were split into two groups of equivalent baseline, which were later exposed to either chronic intermittent ethanol vapor (CIE) or air inhalation. Weeks of CIE or air exposure were alternated with weeks of 2BC four times. On a given week of CIE exposure, mice were placed in inhalation chambers and subjected to four cycles of intoxication (ethanol vapor inhalation for 16 h, from 5:00 PM to 9:00 AM, starting Monday afternoon and ending Friday morning) separated by 8-h periods of withdrawal (air inhalation), and then returned to their home cage for 3 days until 2BC sessions were resumed the following Monday. Weeks of post-vapor (PV) 2BC were conducted in the same way as the baselining (BL) weeks. This paradigm was adapted from previous publications (Becker and Lopez, 2004; Lopez and Becker, 2005).

### Chronic intermittent vapor inhalation

Inhalation chambers (La Jolla Alcohol Research Inc., La Jolla, CA) consisted of standard plastic mouse cages fitted with a Z-shaped wire mesh separation to produce equivalent vapor exposure of two single-housed mice per chamber. Ethanol vapor was created by dripping 95% ethanol into a 2-L Erlenmeyer vacuum flask kept at 50°C on a warming tray. Air was pumped into the flask (HK40L, Hakko, Laguna Hills, CA) and ethanol vapor flew into each sealed chamber at a rate of 3 L/min. Before each onset of ethanol vapor exposure, mice were injected intraperitoneally with a solution of ethanol (1.5 g/kg) and pyrazole (1 mmol/kg, Sigma, St Louis, MO), an alcohol dehydrogenase inhibitor, to initiate intoxication and maintain constant BALs during the 16 h of ethanol vapor exposure. Ethanol vapor concentration was adjusted by varying the ethanol dripping rate, so as to yield BALs approximating 200 mg/dL. Each vapor-exposed mouse was typically sampled twice a week, at the end of a 16-h intoxication period. Blood samples were collected from the tail vein with heparinized capillary tubes and centrifuged for 5 min at 13,000 rpm. The supernatant was processed in a GM7 analyzer (Analox Instruments, London, UK). Mice whose average BAL over the whole experiment was below 170 mg/dL were excluded from data analysis (3 out of 39 mice).

### Data analysis

Statview software (SAS Institute Inc.) was used for statistical analysis. In Experiment 1, ethanol drinking data were analyzed using a Two-Way repeated-measures (RM) analysis of variance (ANOVA) with daily intake as the dependent variable, time as a within-subject factor and genotype as a between-subject factor. Consumptions under continuous (C1 through C15) and intermittent (C15 through I7) access were analyzed separately. In Experiment 2, baseline drinking (intake on last day) was analyzed by One-Way ANOVA with genotype as a between-subject factor, while the effect of ethanol vapor exposure was analyzed in each genotype by Two-Way RM-ANOVA with weekly average intake as the dependent variable, time as a within-subject factor and treatment as a between-subject factor. Average intake on a given PV week was also analyzed with unpaired *t*-tests to evaluate differences between genotype and treatment groups. The data are expressed as mean ± s.e.m. in all graphs.

## Results

### BK β1 and β4 subunits do not influence continuous ethanol drinking

**Figure 1:**
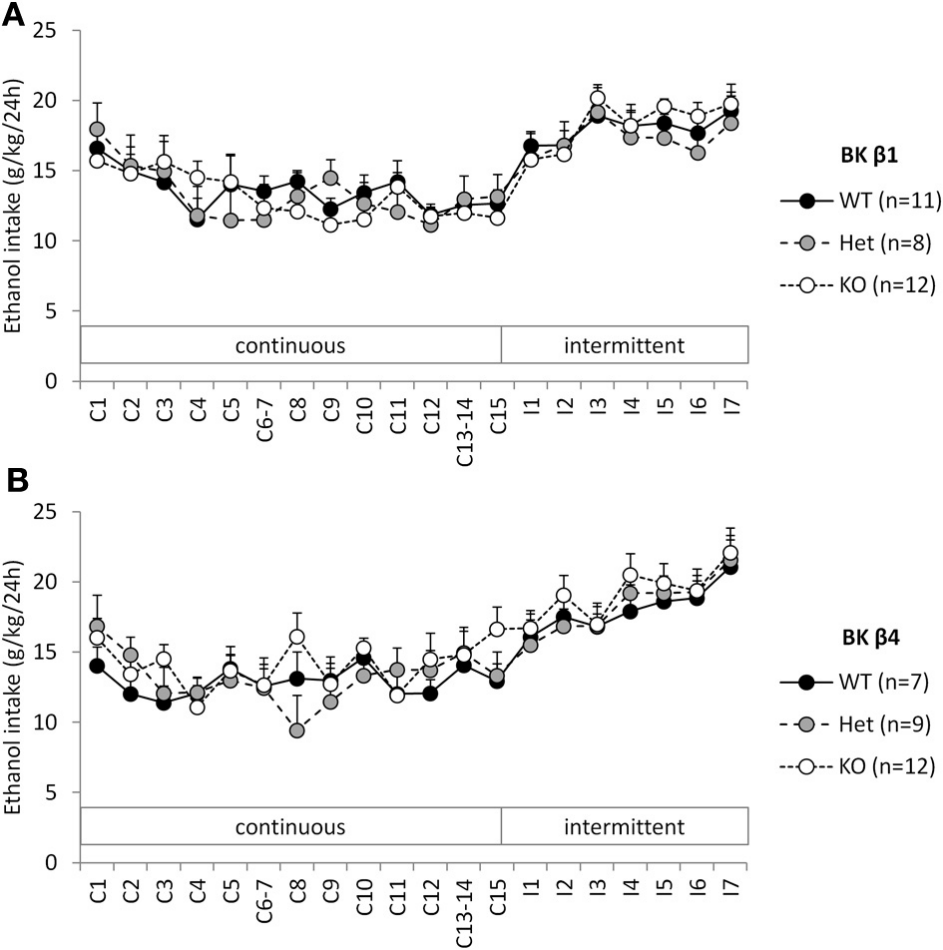
**Experiment 1: Ethanol intake under continuous and intermittent access**. Individually-housed BK β1 **(A)** and BK β4 **(B)** WT, Het, and KO mice were first offered continuous access to ethanol (20% w:v) and water in their home cages for 2 weeks (C1–C15). Access was then switched to intermittent (24-h periods, 3 days a week, I1–I7). Number of mice in each group is indicated in the legend.

When given continuous access to ethanol in their home cage, WT mice stabilized their intake ~12–13 g/kg/24 h (Figures 1A,B). A Two-Way repeated-measure ANOVA detected an effect of time in both strains reflecting the initial intake decline (Table 1). The effect of genotype and the time × genotype interaction, however, were not significant.

**Table 1:** **Statistical analysis of the effects of time and genotype on ethanol intake in Experiment 1**.

**Experiment 1**	**Continuous access**		**Intermittent access**	
**RM-ANOVA**	**BK β1**	**BK β4**	**BK β1**	**BK β4**
Effect of time	*F*_(12, 336)_ = 4.90, *p* < 0.001	*F*_(12, 300)_ = 2.17, *p* < 0.05	*F*_(7, 196)_ = 24.59, *p* < 0.001	*F*_(7, 175)_ = 21.70, *p* < 0.001
Effect of genotype	*F*_(2, 28)_ = 0.04, n.s.	*F*_(2, 25)_ = 0.39, n.s.	*F*_(2, 28)_ = 0.12, n.s.	*F*_(2, 25)_ = 0.36, n.s.
Time × genotype interaction	*F*_(24, 336)_ = 1.00, n.s.	*F*_(24, 300)_ = 1.20, n.s.	*F*_(14, 196)_ = 0.90, n.s.	*F*_(14, 175)_ = 0.78, n.s.

### BK β1 and β4 subunits do not influence intermittent ethanol drinking

When switched to an intermittent schedule of access to ethanol, WT mice progressively increased their intake up to ~19–21 g/kg/24 h after 7 sessions (Figures 1A,B). Accordingly, there was a significant effect of time, but there was again no effect of genotype, nor an interaction between time and genotype (Table 1).

### BK β1 and β4 subunits do not influence limited-access ethanol drinking

**Figure 2:**
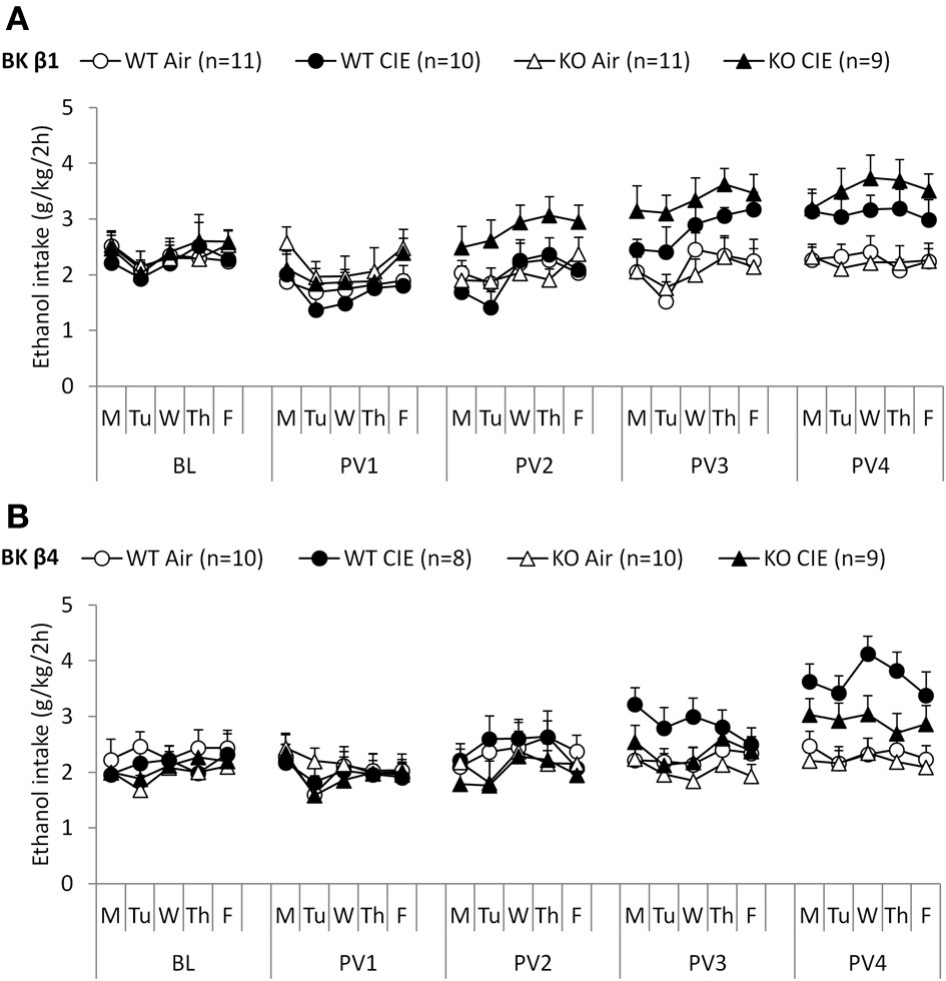
**Experiment 2: Ethanol intake under limited access and following dependence induction**. Individually-housed BK β1 **(A)** and BK β4 **(B)** WT and KO mice were first offered access to ethanol (15% v:v) and water in their home cages for 2 h per day on weekdays (baseline, BL). Weeks of chronic intermittent ethanol (CIE) or air inhalation were then alternated with weeks of post-vapor (PVn) voluntary ethanol drinking. Number of mice in each group is indicated in the legend.

When given access to ethanol 2 h per day at the beginning of the dark cycle, mice stabilized their intake ~2–2.5 g/kg (Figures 2A,B, BL week). Deletion of the BK β1 or β4 subunits again did not affect ethanol drinking under these conditions, as indicated by a One-Way ANOVA (BK β1: *F*_(1, 39)_ = 1.66, n.s.; BK β4: *F*_(1, 35)_ = 0.66, n.s.). Likewise, air-exposed BK β1 and β4 KO mice did not differ from their WT counterparts during any of the subsequent 2BC weeks (Table 3).

### BK β1 deletion accelerates the escalation of ethanol drinking in dependent mice

Repeated exposure to cycles of forced ethanol intoxication and withdrawal through the CIE procedure gradually increased voluntary ethanol drinking in BK β1 WT mice, while air-exposed counterparts maintained a stable intake throughout the experiment (Figure 2A, PVn weeks). Accordingly, a Two-Way RM-ANOVA detected a significant interaction between time and treatment (Table 2). CIE-exposed BK β1 KO mice increased their intake even more robustly, as attested by a stronger interaction between time and treatment, as well as a significant effect of treatment (Table 2). Noticeably, the ethanol consumption of CIE-exposed BK β1 KO mice started rising earlier than for their WT counterparts. Further analysis of intake during PV2 and PV3 detected a significant effect of treatment in KO mice but not in WT mice (Table 3). In addition, the ethanol intake of CIE-exposed BK β1 KO mice during PV2 was significantly higher than the intake of their WT counterparts (Table 3).

**Table 2:** **Statistical analysis of the effects of time and treatment on ethanol intake in each genotype in Experiment 2**.

**Experiment 2**	**BK β1**		**BK β4**	
**RM-ANOVA**	**WT**	**KO**	**WT**	**KO**
Effect of time	*F*_(4, 76)_ = 7.71, *p* < 0.001	*F*_(4, 72)_ = 5.14, *p* < 0.01	*F*_(4, 64)_ = 8.15, *p* < 0.001	*F*_(4, 68)_ = 3.02, *p* < 0.05
Effect of treatment	*F*_(1, 19)_ = 1.09, n.s.	*F*_(1, 18)_ = 5.59, *p* < 0.05	*F*_(1, 16)_ = 1.88, n.s.	*F*_(1, 17)_ = 0.41, n.s.
Time × treatment interaction	*F*_(4, 76)_ = 3.25, *p* < 0.05	*F*_(4, 72)_ = 7.43, *p* < 0.001	*F*_(4, 64)_ = 6.14, *p* < 0.001	*F*_(4, 68)_ = 2.36, n.s.

**Table 3:** **Statistical analysis of the effects of treatment and genotype on ethanol intake during each 2BC week in Experiment 2**.

**Experiment 2**	**BK β1**					**BK β4**				
***t*-test**	**BL**	**PV1**	**PV2**	**PV3**	**PV4**	**BL**	**PV1**	**PV2**	**PV3**	**PV4**
WT Air vs. WT CIE	n.s.	n.s.	n.s.	n.s.	*p* < 0.05	n.s.	n.s.	n.s.	n.s.	*p* < 0.001
KO Air vs. KO CIE	n.s.	n.s.	*p* < 0.05	*p* < 0.01	*p* < 0.01	n.s.	n.s.	n.s.	n.s.	*p* < 0.05
WT Air vs. KO Air	n.s.	n.s.	n.s.	n.s.	n.s.	n.s.	n.s.	n.s.	n.s.	n.s.
WT CIE vs. KO CIE	n.s.	n.s.	*p* < 0.05	n.s.	n.s.	n.s.	n.s.	n.s.	n.s.	*p* < 0.05

### BK β 4 deletion attenuates the escalation of ethanol drinking in dependent mice

BK β4 WT mice likewise increased their intake following CIE exposure, albeit at a faster pace than BK β1 WT mice (Figure 2B, PVn weeks). Accordingly, the time × treatment interaction was strong (Table 2). In contrast, the escalation of ethanol consumption was modest in CIE-exposed BK β4 KO mice, as reflected by the lack of time × treatment interaction in this genotype (Table 2). Further analysis of intake during PV4 confirmed that the effect of treatment was less pronounced in KO mice than in WT mice, and revealed that CIE-exposed BK β4 KO mice drank significantly less ethanol than their WT counterparts (Table 3).

## Discussion

The present study tested whether BK β1 and β4 subunits contribute to voluntary ethanol drinking in different paradigms, each emulating a specific consumption pattern and motivational drive. Continuous access to ethanol leads to sporadic bouts of drinking and produces fluctuating BALs with brief peaks of intoxication (as defined by BALs above 80 mg/dl), but intake under these conditions may not be entirely driven by the pharmacological effects of ethanol (Dole and Gentry, 1984). Conversely, intermittent and limited access to ethanol lead to binge drinking and produce BALs that positively correlate with intake in C57BL/6J mice (Supplementary Figure S1, Becker and Lopez, 2004; Rhodes et al., 2005; Hwa et al., 2011; Contet et al., 2013). Intake during the binge episode depends on its timing within the light-dark cycle (Rhodes et al., 2005). When occurring few hours into the circadian dark phase (as in the intermittent access model used in the present study), it consistently produces significant intoxication (Supplementary Figure S1, Hwa et al., 2011; Contet et al., 2013). In contrast, if the binge episode occurs at the very beginning of the dark phase (as in the limited access model used in the present study), consumption is lower and BALs are most often not pharmacologically significant (Becker and Lopez, 2004). Dependence induction through CIE exposure however increases the rate of ethanol intake during such limited-access drinking sessions, and accordingly increases blood and brain ethanol concentrations beyond intoxicating levels (Becker and Lopez, 2004; Griffin et al., 2009). Most importantly, voluntary drinking in dependent rodents (i.e., following CIE exposure) is driven by negative reinforcement rather than by the pleasurable effects of ethanol intoxication [see Koob (2003); Gilpin and Koob (2008) for reviews].

Our results indicate that the absence of BK β1 or β4 subunits doesn't impact voluntary ethanol consumption unless the mice are dependent. In particular, we didn't observe an effect of BK β1 or β4 deletion on binge drinking in either the intermittent or limited-access paradigms. The present observation is at odds with the increased ethanol intake of BK β4 KO mice that was previously reported in the “drinking-in-the-dark” model (Martin et al., 2008). In the latter study, mice had access to a single bottle of ethanol during 2-h sessions scheduled 2 h after lights out. The unavailability of water at a circadian time of high fluid consumption may have resulted in ethanol intake driven by homeostatic needs rather than by hedonic effects, thereby differing from our 2BC paradigms. Another important discrepancy relates to the control mice. Martin et al. bred KO mice in their facility but independently obtained C57Bl/6J mice from Jackson Laboratories. In contrast, we used WT and KO littermates, which better controls for raising conditions and other potential artifacts. Altogether, our results support the hypothesis that BK β1 and β4 do not modulate binge drinking.

Importantly, dependence-induced escalation of voluntary ethanol consumption was altered in BK β1 and β4 KO mice. This finding suggests that chronic ethanol intoxication somehow recruits BK auxiliary subunits. It is noteworthy that the absence of BK β1 had significant behavioral consequences, although this subunit has been shown to be expressed at very low levels in the brain of naïve mice (Chang et al., 1997; Jiang et al., 1999; Behrens et al., 2000; Brenner et al., 2000). The presence of β1 in neurons has however been inferred from the existence of ethanol-insensitive BK channels in nucleus accumbens and hypothalamic neurons, as well as from positive immunostaining (Martin et al., 2004; Wynne et al., 2009). Our data suggest that CIE exposure may induce higher levels of BK β1 expression, at least in local brain regions or specific neuronal populations.

Most interestingly, BK β1 and β4 appear to exert opposite influences on the voluntary ethanol intake of dependent mice, as BK β1 deletion accelerated and BK β4 attenuated the escalation of drinking in CIE-exposed mice. The divergent phenotypes of BK β1 and β4 KO mice could result from the differential role of the two subunits in the development of molecular tolerance to ethanol. Since the association of β1 has been shown to abolish the sensitivity of BK currents to ethanol-induced potentiation (Martin et al., 2004; Feinberg-Zadek and Treistman, 2007), the putative recruitment of BK β1 would dampen the effect of ethanol on BK channels. Recruitment of BK β1 could also reduce ethanol-induced potentiation by regulating the surface expression of BK α isoforms exhibiting differential ethanol sensitivity (Toro et al., 2006; Kim et al., 2007). The absence of BK β1 may prevent such desensitization mechanisms from being engaged in chronically intoxicated BK β1 KO mice, which could trigger the onset of cellular adaptations offsetting the prolonged and excessive stimulation of BK channels by ethanol. These adaptations could facilitate some of the neuroplastic changes underlying the escalation of ethanol intake in dependent animals (Koob, 2003; Gilpin and Koob, 2008), thereby accelerating the increase in voluntary ethanol consumption of CIE-exposed BK β1 KO mice. Conversely, BK β4 has been shown to oppose the development of acute tolerance to the effects of ethanol on BK channel gating, neuronal excitability, and locomotor activity (Martin et al., 2008). The actual mechanism through which β4 controls desensitization remains to be elucidated, but it could involve the capacity of β4 to modulate the trafficking (Shruti et al., 2012) and phosphorylation status (Petrik et al., 2011; Gilles Martin, personal communication) of BK α in a palmitoylation-dependent manner (Chen et al., 2013). In any case, in the absence of BK β4, ethanol-induced potentiation of BK currents is very short-lived, which may attenuate the development of the above-mentioned counteradaptations, thereby limiting the escalation of voluntary ethanol drinking in CIE-exposed BK β4 KO mice.

Since constitutive KO mice were used in the present study, we cannot exclude that the phenotypes we observed in dependent mice result from a compensatory upregulation of β1 in BK β4 KO mice or β4 in BK β1 KO mice. It should be noted, however, that BK β1 expression in the hippocampus is unaffected by BK β4 deletion (Petrik et al., 2011). Another factor that could explain the opposite influence of BK β1 and β4 on ethanol drinking in dependent mice relates to their differential distributions within brain regions (Chang et al., 1997; Jiang et al., 1999; Behrens et al., 2000; Brenner et al., 2000), neuronal populations (Sugino et al., 2006), and subcellular compartments (Martin et al., 2004; Wynne et al., 2009). Future studies aiming at silencing each subunit in local brain regions of adult animals will help better delineate the role of BK channel auxiliary subunits in the behavioral manifestations of ethanol dependence. Bearing these caveats in mind, our results provide promising evidence that the modulation of BK channel properties can represent a successful strategy to selectively alter the motivational drive of dependent subjects to drink ethanol in excess.

### Conflict of interest statement

The authors declare that the research was conducted in the absence of any commercial or financial relationships that could be construed as a potential conflict of interest.

## Supplementary material

Figure S1 **Intermittent access to ethanol drinking produces intoxicating blood alcohol levels.** BALs were measured in C57Bl/6J mice offered continuous or intermittent access to ethanol (20% w:v) along with water. Tail blood was sampled 1 h **(A)** or 24 h **(B)** after the ethanol bottle was reintroduced (intermittent) or repositioned (continuous). Linear regression analysis detected a significant correlation between BAL and ethanol intake when blood was sampled 1 h after ethanol bottle reintroduction in mice given intermittent access [*R*^2^ = 0.66, *F*_(1, 5)_ = 7.61, *p* = 0.05, dashed line], but not under other circumstances. In the former condition, BALs were above the intoxication threshold (80 mg/dL) in all mice tested.
