# Supplementary figures and images for: BK channel β1 and β4 auxiliary subunits exert opposite influences on escalated ethanol drinking in dependent mice
Source: Front Integr Neurosci. 2013 Dec 30;7:105. doi: 10.3389/fnint.2013.00105 (PMC3874544; doi:10.3389/fnint.2013.00105)

# Supplementary Figure S1

**A**

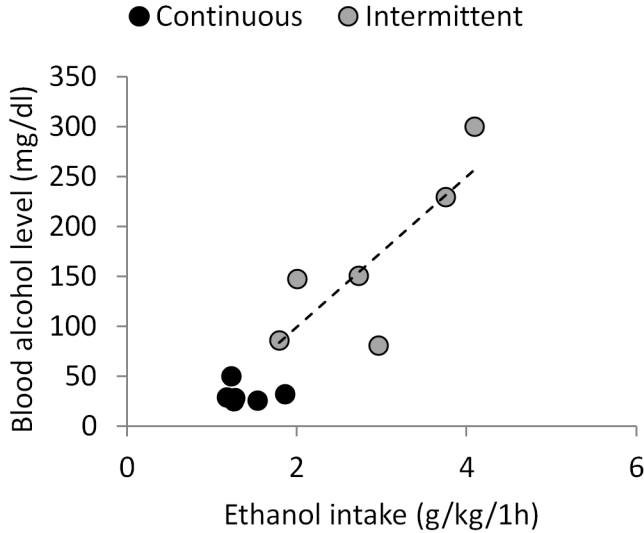

**B**

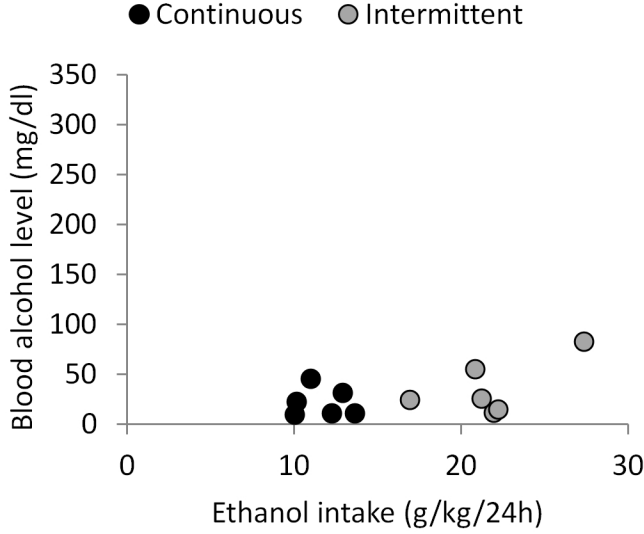

Supplement: Figure S1 — Intermittent access to ethanol drinking produces intoxicating blood alcohol levels. BALs were measured in C57Bl/6J mice offered continuous or intermittent access to ethanol (20% w:v) along with water. Tail blood was sampled 1 h (A) or 24 h (B) after the ethanol bottle was reintroduced (intermittent) or repositioned (continuous). Linear regression analysis detected a significant correlation between BAL and ethanol intake when blood was sampled 1 h after ethanol bottle reintroduction in mice given intermittent access [R2 = 0.66, F(1, 5) = 7.61, p = 0.05, dashed line], but not under other circumstances. In the former condition, BALs were above the intoxication threshold (80 mg/dL) in all mice tested. [file Presentation1.PDF]
